# Supplementary figures and images for: Engineering selectivity of Cutibacterium acnes phages by epigenetic imprinting
Source: PLoS Pathog. 2022 Mar 28;18(3):e1010420. doi: 10.1371/journal.ppat.1010420 (PMC8989293; doi:10.1371/journal.ppat.1010420)

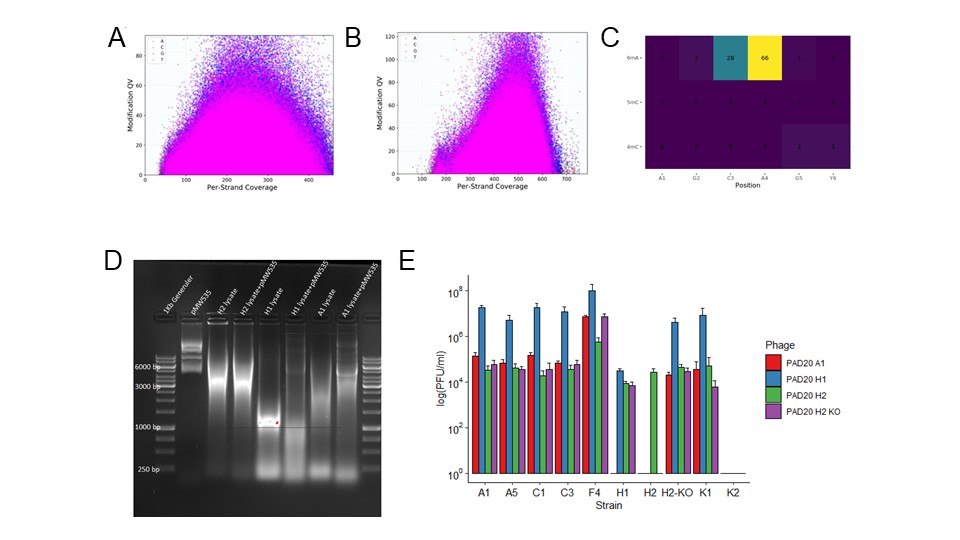

Supplement: S1 Fig — (A) (B) Distribution of the Modification QV value from SMRT sequencing across different coverage regions for SLST H1 (A) and SLST A1 (B) samples. No clear enrichment is shown for any of the bases, indicating the lack of a methylation motif. (C) Predicted modification for the different positions within the AGCAGY motif according to the multi-label classification algorithm. Most modifications were classified as 6mA and located in the 4th adenine of the motif. (D) Restriction profiles of plasmid DNA incubated with lysates of C. acnes SLST H2 (KPA171202), H1 and A1. Lane 1 and 9 molecular weight markers 1 kb GeneRuler (Thermo Fisher); Lane 2, plasmid control pMW535 1ug; Lane 3, H2 lysate; Lane 4, H2 lysate+1ug pMW535; Lane 5, H1 lysate; Lane 6, H1 lysate+1ug pMW535; Lane 7, A1 lysate; Lane 8, A1 lysate+1ug pMW535 (E) PAD20 phage infection properties on different C. acnes strains depending on propagation origin (SLST A1, H1, H1 or H2 KO). (E) Infection of a selection of C. acnes strain belonging to different clades with PAD20 propagated on SLST A1, H1, H2 and H2 KO. Phages acquire different infection properties depending on their origin. (TIF) [file ppat.1010420.s001.tif]

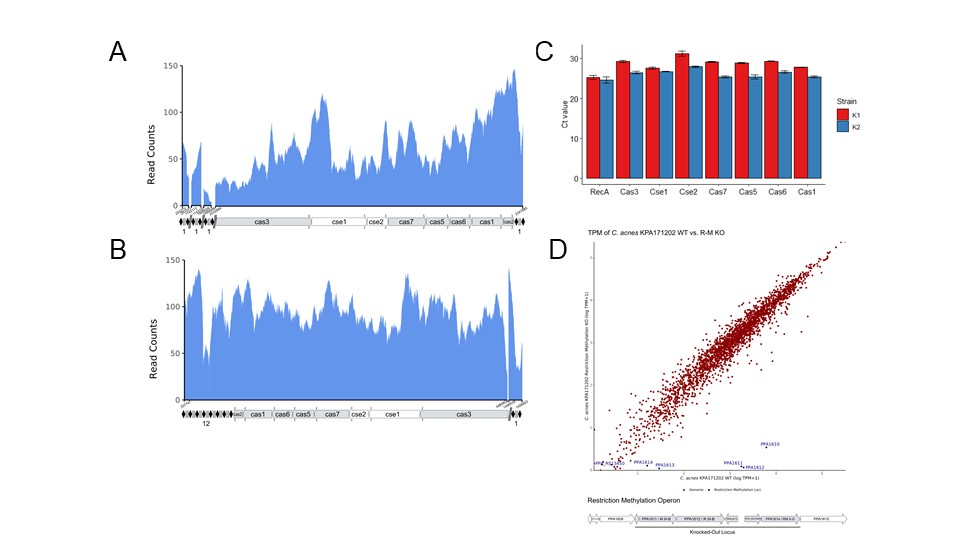

Supplement: S2 Fig — Transcriptional profile and architecture of CRISPR/cas type I-E locus found in (A) C. acnes SLST K1 (09–9) and (B) SLST K2. Expression levels are displayed by RNA-seq read counts per genomic position. Black diamonds and grey squares represent CRISPR clusters, with the number of total repeat-spacer-repeats indicated below. (C) Expression levels of genes associated with the CRISPR cascade in C. acnes SLST K1 (blue) and K2 (red) determined by qRT-PCR across 3 independent replicates per strain and gene. RecA was used as control for a housekeeping gene. Displayed are threshold cycles (CT) and were used to classify gene expression as high (21–25 CT), Moderate (26–30 CT) or Low (31–38 CT). Error bars indicate standard error. (D) Top panel: Expression levels in C. acnes KPA171202 Wild Type (SLST H2 WT) and restriction methylation locus knockout (SLST H2 R-M KO) displayed by logarithm of RNA-seq transcripts per million plus one (TPM+1) (mean of two replicates) per gene. Bottom panel: Restriction methylation operon architecture showing knocked out loci. (TIF) [file ppat.1010420.s002.tif]

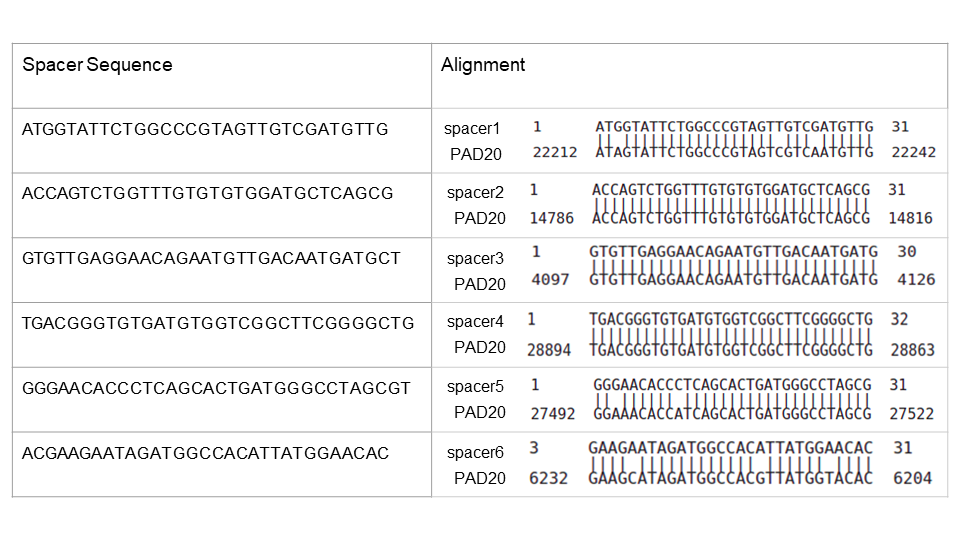

Supplement: S1 Table — (TIF) [file ppat.1010420.s003.tif]
