# Supplementary material for: Engineering selectivity of Cutibacterium acnes phages by epigenetic imprinting
Source: PLoS Pathog. 2022 Mar 28;18(3):e1010420. doi: 10.1371/journal.ppat.1010420 (PMC8989293; doi:10.1371/journal.ppat.1010420)
Supplement: Attachment — Submitted filename: response to reviewers.pdf [file ppat.1010420.s006.pdf]

Revision Plan

Manuscript number: RC-2021-00950
Corresponding author(s): Güell, Marc

1. General Statements [optional]

We thank the reviewers for their insightful comments. See below for specific actions taken and in the process of being taken. We request an extension of 6 weeks to include all new revisions (specially the RNAseq part has a ~6-week turn-around).

2. Description of the planned revisions ([redacted])

3. Description of the revisions that have already been incorporated in the transferred manuscript (in green)

Reviewer #1 (Evidence, reproducibility and clarity (Required)):

In this manuscript, authors surveyed prevalence of the R-M systems in the selected C. acnes strains using the PAD20 phage. They screened the methylation patterns in their genomes using the Oxford Nanopore technologies in order to find some correlation between the epigenetic status and phage DNA restriction observed in the experimental approach. The authors showed that the specific methylation in one strain exerts the variation on phage susceptibility in other strains while phage transfers from one strain to another. They did all necessary experiments to underpin their thesis.
Overall, I find this report hard to follow due to many unclear spots in the text that often are not linked properly to the Figures (mainly at results section).
##Thank you for highlighting this, we have re-written passages in the results, have divided them in subsections instead of having a continuous text and have properly linked them to figures to better understand the results.

Major points:

I find not clear why the authors used the cell lysis spectrum parameter to test the infectivity of selected strains (heat-maps) by PAD20 phage. Usually shaping this characteristic quantitatively as the efficiency of plaquing (EOP) is best approach (e.g. in serial dilutions). It is known facts the R-M system does not provide the perfect protection against the phage, rather it decreases the chance of cell lysis. So numbers/ratios are more important than type of lysis. This should be explained in Method section. Opaque vs. clear plaques may indicate some other phenomenon. Maybe the plaques phenotypes: full / moderate lysis could be added to the supplement.

##Thank you so much for pointing this out. We repeated the assay using the EOP system with serial dilutions and calculated the PFU/mL. Data is now represented in a bar plot instead of a heatmap for increased clarity. (now Figure 2A and Supplementary Figure 1E)

The figure legends seem to be very messy, unclear with neither proper explanation of axes nor panel descriptions, e.g. Figure 2E. The reader should know, not to guess. e.g. A.F.C.prop?????

##We fixed the figure 2E as recommended and tried to clarify overall legend description as indicated. The figure shows the available fractions of SLST reads at the three indicated time points. The similarity to the SLST alles is used to classify sequencing reads into the clades IA, IB and II. (now Figure 2C)

There's contrast or rather error in presentation of R-M systems in Figure 1A and graphical abstract for H1 and H2. Why???

We have modified Figure 1A to have more clarity. The previous figure displayed R-M systems in all genome assemblies available and compared them with each other. The new figure now represents all newly sequenced genomes used in the study and its identified R-M systems.

I also tried to digest the part of data related to Figure 2E. I find this poorly described so I cannot sufficiently appreciate the outcome in the current state. This section should be re-written and effort should be focused to refer more to data from Figure.

We fixed the figure 2E as commented and tried to clarify the overall legend description as indicated and we modified the section in the main text. (Now Figure 2C; see Line 237-249)

Minor points:

Page 2 and elsewhere, follow the R-M system nomenclature, type IIB, type IIIB etc.
Thank you for indicating. We have checked the text and corrected the nomenclature of the R-M systems throughout the text.

Page 4. The information on SLST and C. acne types should be explained more, so that the reader is not lost in following text sections.
Thank you for pointing this out, we have added a paragraph on the different typings available describing C. acnes phylogenetic types. (see line 116-120)

Page 5. The results initial part is very unclear in its present form. There are too many abbreviations related to phages, R-M systems and strains, so it is very confusing and hard to digest the information. I'd suggest to modify the Figure 1A or graphical abstract, and to extend its legend to explain all necessary symbols and refer to it frequently.
##We understand the nomenclature can be confusing since they are quite similar. We have modified Figure 1A and graphical abstract and added legend descriptions so ease the information given in the text. We also have re-written results to make it easier for the reader

to follow. (e.g. using SLST A1 instead of A1 only, using clade II instead of type II for the phylogenetic relationship of strains to differentiate from R-M systems which are also named IIG for example.)

Page 5. Is PAD20 phage lytic? Any information about its host range or receptor, if known?
##We have added a paragraph on the specifics of PAD20 phage. (see Line 96-102)

E.g. #1 "While most type IA1 and IA2 strains were lysed by all three PAD20 phages, K2 (type II) was not able to be lysed by any of the three even though originally was extracted from clade II". - Type IA2 is not indicated at Figure 1A. Type IA2 and Type II might be confusing.
##In the text we compare C. acnes R-M systems on clade and strain level. We understand that this might lead to confusion since there are so many abbreviations. We hope that the extra information in the figures 1A and 2C makes the relationships more clear and less confusing. Clade IA1 and IA2 were fused to clade IA since those behave similarly in the assay.

E.g. #2 "Type III strain L1 was also highly susceptible by all three PAD20 phages". - Again Type III is not present at the figure. Use the same codes throughout the entire text.
##After repeating the assay using the EOP system we did not have conclusive results with L1 since we could not identify if there was no growth or full lysis. Due to this we left out L1 from the figures and the manuscript.

If H2 bacteria had inactive R-M system, which system specified the AGCAGY sequence methylation pattern.
Figure 1A. More comment in the legend related to R-M system presence, specify MTase, REase, hsdR/M/S etc. Inactive R-M system in H2 or H1??? Is it error? Specify arrow, active vs inactive R-M system.
##Thank you for letting us know. Figure 1A was used as a summary for different assemblies of strains of the same SLST type. While some H1 have active systems our sequenced strain has a broken ORF and seems therefore to be inactive. On the other hand, H2 has a complete frame of MTase and REase and showed the AGCAGY methylation sequence. We agree it might have been very confusing and complicated to follow so we have changed figure 1A to only include the frames of the tested strains excluding previously sequenced genome assemblies available on NCBI.

Figure 1B. What was the criterion to determine the cell lysis level and make the heatmap? Full lysis (clear plaques) versus no lysis (no plaques), but how about other in-between/moderate lysis? Any variation in plaque phenotypes for selected strain/phage pairs??? Specify KO in the legend. The KO strain might be added to Figure 1A for clarity.

##Phage lysis properties were determined by degree of proceeded lysis. The heatmap displays lysis by giving lysis potential a number 0-4 where 0 is no lysis and 4 is full lysis of

plate. To increase clarity we repeated the experiment using the EOP system with a set phage titer and represented PFU/mL in a bar plot. We also have added H2 KO in figure 1A for clarity.

Page 5 "Interestingly, a S-adenosyl synthase is located just upstream of the methylase gene". A word "gene" is missing.
##Thank you, the word has been added. (see Line 127)

Page 7 In the methylation motif, G is missing. "motif (AGCA(6mA)GY)"
##Thank you, we included the G in the motif. (see Line 158)

Page 8, Mention the number of AGCAGY sites in the phage PAD20 genome.
##We have mentioned in Page 9. PAD20 has 20 AGCAGY motifs in its genome sequence. (see Line 223)

Page 14, "subset of colonies.. by SLST sequencing". How many roughly? 20, 50 or 100??
##Sorry, we haven't mentioned it before. We have tested 10 single colonies. (see Line 234)

Reviewer #1 (Significance (Required)):

See above.

Reviewer #2 (Evidence, reproducibility and clarity (Required)):

Summary:

This study performs a first epigenetic study on C. acnes using long read sequencing technologies. The authors map different types of putative R-M systems and orphan methyltransferases, and test for their functionality. They next test for the interplay between methylome and resistance to phage infection.

Major comments:

This is an interesting study, conceptually and experimentally well supported. Unfortunately, as currently is, this manuscript only pinpoints one active R-M across C.acnes, and verifies that it offers anti-phage protection. These observations lack substantial novelty, and also fall short in their ability to, as stated in the Conclusion section by the Authors, provide a more holistic view of the defensome. Such additions would provide the reader with a more complete and understandable view of the arsenal of tools regulating gene-flux in C. acnes. This becomes particularly important under a scenario in which just one R-M system was found as functional, and already described in the literature). A few examples on how to improve the general interest and strength of the manuscript are provided below.

Minor comments:

1) The Authors mention at some point "A reason for this observation could be the existence of CRISPR spacers against PAD20 phages...(data not shown)". CRISPR-Cas mapping and spacer-phage-homology analyses would be very straightforward and quick to perform.

##We analyzed spacers against PAD20 Phage in K2 strain. We have provided the data in Supplementary Table 1.

##We also added expression data of the CRISPR/Cas type I-E systems which show expression of the complete cascade in K1 and K2 strain (clade II). (Both RT-qPCR and RNAseq) (Figure 2A,B,C)

2) On top of this, and since the Authors are dealing with such a small number of genomes, it would be also be very quick to perform a mapping of the number (or density) of other defense systems. For this, they could use dedicated HMM profiles that are already available (BREX, TAs, DISARM, Argo-PIWI, DRUANTIA, GABIJA, ZORYA). This would take 3-4 days of work, and would "provide a full picture on how these bacteria are defending from HGT.

We used HMM profiles available in PFAM as well as a dedicated tool called DefenseFinder to search for additional defense systems in *C. acnes*. Although hits for nearly all systems were observed, there was no evidence of a complete set of genes for any of them. The only defense systems identified with significant hits are summarized in the figure below where each gene is named after the HMM model name.

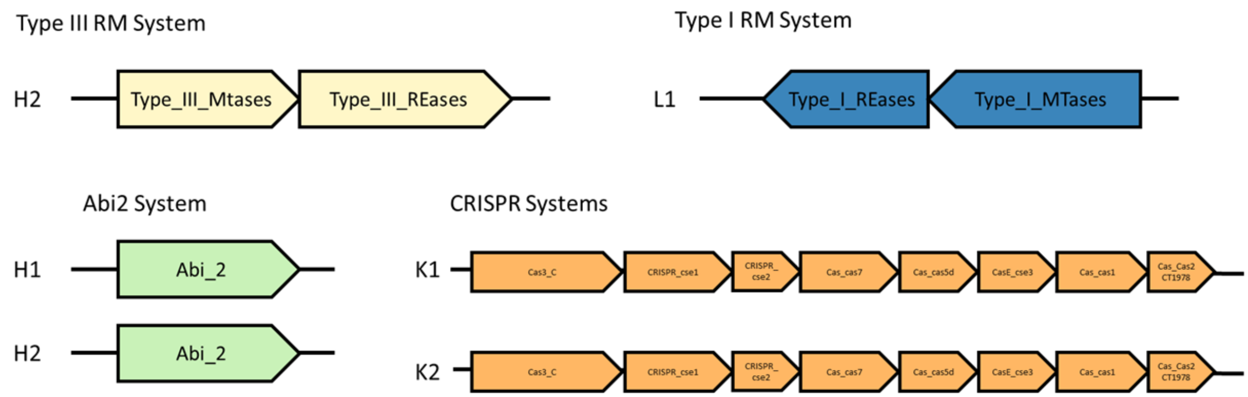

3) If I well understood, the Authors did not map R-Ms and orphan MTases in the phage genomes? This would be of interest, as it would show how the latter are adapting to host infection and arms-race.

A search using dedicated HMM models from well-known methylase domains annotated in PFAM did not yield any significant hit in the PAD20 genome. We also checked for potential remnants of other phage-defense systems but again there were no significant hits.

4) Background: "90% of sequenced bacterial genomes harness a R-M system [2]." Please change harness to harbor. Also, it would be unfair for the following authors not to be cited here:

https://pubmed.ncbi.nlm.nih.gov/25120263/
https://journals.plos.org/plosgenetics/article?id=10.1371/journal.pgen.1005854

##Thanks for pointing this out. We have added these references and have changed harness to harbor throughout the text. (see Line 53)

5) "Understanding R-M is key to...". Understanding what? R-M functionality? Diversity? Please spec

##Thank you for this comment. We have specified in the text. (see Line 60-63)

6) "The stability of the genome may lead to the conclusion that C. acnes harbours multiple defence strategies which would not allow genetic changes." -> This is a dangerous sentence. As seen previously, more systems do not always translate into better protection. We have to take into consideration systems' compatibility between host and invader. For example, if a bacterial host has 3 (high number) R-Ms with different target sequences, and a phage has 3 orphan MTases with the same target site, they will be compatible, and gene flux will be high.

##Thank you! That is completely correct. We have explained concretely what we meant by this sentence. (see Line 81-83)

7) It was weird to me to see a Suppl Table 3 with such a large number of acnes strains, but for which only 7 were effectively used in this study. Why did the authors didn't extend the analysis to the whole set (to increase the confidence of conclusions)?

##We have used genome assemblies for homology and HMM comparison reasons but then chose a representative selection of each clade for identification of functional R-M systems by ONT sequencing since frames were identical.

8) Fig. 1B. How was lysis extent translated into a heatmap value?

##Proceeded Lysis and lysis potential of different phage extractions were numbered between 0-4, where 0 is no lysis detected and 4 is full lysis of plate. We have repeated this experiment by using the EOP system and represented PFU/mL in a bar plot instead of a heatmap.

9) I did not find in the materials and methods the protocol for SMRT-seq confirmation.

##We have added a section in the method. Library prep and sequencing were done by the Norwegian sequencing center. (see Line 453-458)

10) Wouldn't an HMM search (instead of homology search) yield higher confidence in R-M detection? HMM profiles are available.

We used HMMER 3.3 and models available from PFAM as well as a dedicated tool, DefenseFinder (based on Macsypy) to validate the search for R-M systems. Specifically, the type III RM system from H2 was a clear significant hit to ResIII and N6_N4_Mtase family models (E-value for restriction and methylase domains both less than 10^{-10})

11) "However, it remains unclear which variant expresses a functional R-M system or if neither of them is functional." -> Couldn't this be done with ONT or Pacbio data?
##We have clarified and changed this sentence. (see Line 145-146)

12) The cerise on top of the cake would be to have the functional role of this MTase. Which would mean, doing some RNA-seq on WT versus MTase mutant. This would certainly bring the paper to a higher level.
##Thank you for this interesting extra. We performed RNAseq on the WT and KO genome and analyzed the differences. Indeed, the only differences we could identify are the genes targeted for the KO (see Figure 1E and Supplementary Figure 2D). It does not seem that there is an additional function of the MTase. (also see Line 184-190)

Reviewer #2 (Significance (Required)):

Although several improvements are in my opinion required, this work provides a significant contribution to the expanding field of bacterial methylomics.

Since this is a pre-publication, I opt to disclose my identity in case the Authors would like to discuss further on any of these topics.

Best regards,

Pedro H. Oliveira
Genoscope

Referee Cross-commenting

I agree with Reviewer's 1 assessment of the manuscript.

4. Description of analyses that authors prefer not to carry out

We are planning to address all comments.
